# AI ethics with Chinese characteristics? Concerns and preferred solutions in Chinese academia

**DOI:** 10.1007/s00146-022-01578-w

**Published:** 2022-10-17

**Authors:** Junhua Zhu

**Affiliations:** Centre for East Asian Studies, University of Turku, Turku, Finland

**Keywords:** AI ethics, China, Academic discourse, Systematic review

## Abstract

Since Chinese scholars are playing an increasingly important role in shaping the national landscape of discussion on AI ethics, understanding their ethical concerns and preferred solutions is essential for global cooperation on governance of AI. This article, therefore, provides the first elaborated analysis on the discourse on AI ethics in Chinese academia, via a systematic literature review. This article has three main objectives. (1) to identify the most discussed ethical issues of AI in Chinese academia and those being left out (the question of “what”); (2) to analyze the solutions proposed and preferred by Chinese scholars (the question of “how”); and (3) to map out whose voices are dominating and whose are in the marginal (the question of “who”). Findings suggest that in terms of short-term implications, Chinese scholars’ concerns over AI resemble predominantly the content of international ethical guidelines. Yet in terms of long-term implications, there are some significant differences needed to be further addressed in a cultural context. Further, among a wide range of solution proposals, Chinese scholars seem to prefer strong-binding regulations to those weak ethical guidelines. In addition, this article also found that the Chinese academic discourse was dominated by male scholars and those who are from elite universities, which arguably is not a unique phenomenon in China.

**Supplementary Information:**

The online version contains supplementary material available at 10.1007/s00146-022-01578-w.

## Introduction

At World AI Conference in 2019, Elon Musk and Jack Ma expressed two contrasting attitudes when they had a debate on AI. The former saw AI as the biggest existential threat to humanity while the latter suggested embracing the technology for a brighter future. Accompanying the proliferation of such debate in different domains, in recent years AI ethics has become a global theme of discussion and research (Mittelstadt 2019). Accordingly, academic literature on AI ethics has boomed. Ethics committees and guidelines have also emerged in both private companies and policy circles. Although those progresses have contributed to the general identification and conceptualization of ethical risks of AI, they remain far from sufficient for ethical AI development. The insufficiency is triple-folded. First, notwithstanding a seemingly global convergence on some key principles such as transparency, justice and fairness, non-maleficence, responsibility, and privacy, sustainability- and solidarity-related principles are missing from those guidelines (Jobin et al. 2019). Further, focus has been on the formation of those guidelines rather than transformation into real actions, keeping the effects in check (McNamara et al. 2018; Jobin et al. 2019). In particular, private companies and governments are suspected of ethics washing, meaning issuing non-binding ethical guidelines as a portmanteau to eschew regulation (Wagner 2018; Jobin et al. 2019; Rességuier and Rodrigues 2020). Finally, yet importantly, due to different interests or cultural and political contexts, AI can have distinct ethical implications and social impacts depending on the region. Even though advocating same principles, the prescribed methodologies may differ greatly among different societies (Fung and Etienne 2021). Yet the current literature on AI ethics is in lack of research focusing on the regions outside the West, restraining diversity and inclusivity in terms of academic debates, as well as hampering the global governance of AI (Hagerty and Rubinov 2019; ÓhÉigeartaigh et al. 2020).

According to *The AI Index 2021 Annual Report* from Stanford University, China now leads in both the total number of AI journal publications and the AI journal citations worldwide (Zhang et al. 2021). However, given the statistics from *AI Ethics Guidelines Global Inventory*, China has far fewer domestic guidelines on AI ethics than the EU and the USA. Does this mean that in China, attention has been paid only to the research and development of AI instead of the ethics? In fact, latest studies have shown that in China, there are extremely lively and diverse discussions about social and ethical implications of AI on social media platforms (Mao and Shi-Kupfer 2021); and that China’s AI ethics and governance landscape is shaped by multiple actors including government, private companies, academia and the public (Arcesati 2021). The world needs to shift its attention from whether China is having these discussions to what the substances of the discussions are (Lewis 2018). Moreover, there is a present environment of “AI race” between the USA and China, which may fuel the mistrust and misunderstanding, forming an enormous barrier to international cooperation on the governance of AI (ÓhÉigeartaigh et al. 2020). In this conjuncture, more research on AI ethics in the context of China is in urgent need.

Researchers have recently embarked on decoding the Chinese considerations of AI ethics with different approaches. Some look at them from a rather holistic view. For instance, Rebecca Arcesati produced a comprehensive report on how different stakeholders have shaped the discussions in China (Arcesati 2021); Roberts et al. analyzed the Chinese ethical concerns about AI under an overall framework of strategic policymaking (Roberts et al. 2020). On the other hand, some focus closely on the specific domains, especially online public opinion (Zeng et al. 2020; Mao and Shi-Kupfer 2021). Nonetheless, analysis on the academic discourse is still missing from the literature. Given the fact that prominent scholars constitute the majority members of those local ethical committees and that Chinese academia also follows the governmental initiative to actively engage in international exchanges on the governance of AI (Arcesati 2021), an elaborate study on Chinese academic discourse is expected to provide more nuanced insights in aspects of the Chinese debates on AI ethics as well as their global implications. Therefore, this article makes the first in-depth examination on the current state of discourse on AI ethics in Chinese academia, via a systematic review. This article has three main objectives: (1) to identify the most discussed ethical issues of AI in Chinese academia and those being left out (the question of “what”); (2) to analyze the solutions proposed and preferred by Chinese scholars (the question of “how”); and (3) to map out whose voices are dominating and whose are in the marginal (the question of “who”).

From a perspective of global governance, knowing and understanding the debates *there* is essential for less dystopia-utopia dichotomic judgements on AI’s impacts on *that* society and more critical thinking on whether tradeoffs between conflicting norms are legitimate and just in *that* culture. This article is proposed to facilitate the process of such knowing and understanding. Section 2 offers an overview of the current landscape of AI ethics research in China and its potential global implications. Section 3 presents the employed methodology, including the selection of literature, the coding and categorization process, and the limitations. Findings are presented in Sect. 4, which serves as the foundation of conclusive discussion in Sect. 5.

## AI ethics with Chinese characteristics? Local landscape and global implications

In the Chinese context, although its AI competence has drawn much attention from the international society, the Chinese discussion on AI ethics is still understudied by foreign scholars. In fact, the government has expressed its firm intention to address ethical issues from AI with the publication of *New Generation Artificial Intelligence Development Plan* in 2017, which calls for research on legal, ethical, and social issues from AI and aims to establish a legislation system and ethical framework ensuring the healthy development of AI (State Council 2017). Hitherto, there are three comprehensive government-backed documents about AI ethics in China, namely *Beijing AI Principles* from Beijing Academy of AI (BAAI), *Joint Pledge on Self-discipline in the AI Industry* from AI Industry Alliance (AIIA), and *Governance Principles for a New Generation of AI: Develop Responsible AI* from Ministry of Science and Technology (MOST). In addition to the five convergent principles, they all listed “*diversity & inclusivity*” and “*open & sharing*” as key principles, stressing the importance of cooperation across disciplines, domains, and borders (AIIA 2019; BAAI 2019; MOST 2019), providing a policy foundation for other stakeholders to join the conversation on how AI should be governed. Domestic scholars have published a great number of articles on the topic (Chen et al. 2020); tech giants have issued ethical documents to guide the development of AI in the industry (for instance, Tencent 2020; SenseTime 2021); media has substantial leverage to drive public discourse (Ouchchy et al. 2020); and even the public, although usually not seen as a decisive force in the policy circles, have actively participated in the online debate (Mao and Shi-Kupfer 2021) and pushed for some regulatory changes (Arcesati 2021).

**Fig. 1:**
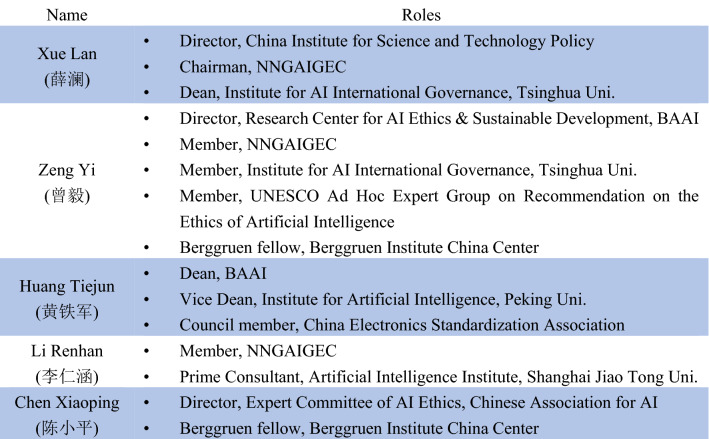
Examples of influential scholars in AI ethics research in China

Scholars, in particular, are playing an increasingly important role in shaping the landscape of AI ethics discussion in China (ibid.). This is well explained by three reasons. First, they are frequently participating in cross-domain debates. For instance, Xiang Biao (项飙), as one of contemporary China’s most renowned anthropologists, has been invited to deliver speeches at Tencent’s Tech for Good Conference, raising concerns about the relations between humans and algorithm systems (Tencent Research Institute 2021). Second, the government aims to increase the country’s “discourse power (话语权)” (Arcesati 2021) and to participate more actively in the rulemaking process of AI development. Therefore, Chinese scholars are following this initiative to actively engage in global exchanges on AI ethics (ibid.). Third, and perhaps most importantly, Chinese scholars occupy the most important positions in those local ethical committees of AI governance. Xue Lan (薛澜), the director of China Institute for Science and Technology Policy, for instance, serves also as the chairman of the National New Generation Artificial Intelligence Governance Expert Committee (NNGAIGEC), which drafted the official governance principles for responsible AI (MOST 2019). Other prominent scholars who have been particularly influential in AI ethics research in China include Zeng Yi (曾毅), Huang Tiejun (黄铁军), Li Renhan (李仁涵), and Chen Xiaoping (陈小平), as Fig. 1 lists.

**Chart 1:**
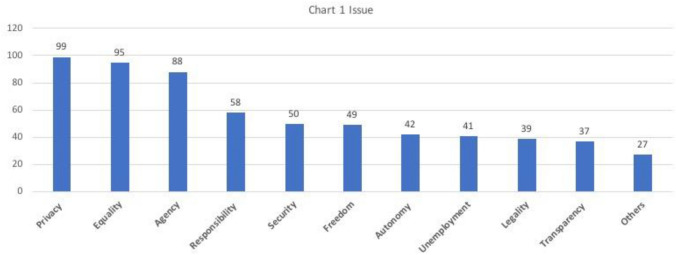
Issue

**Chart 2:**
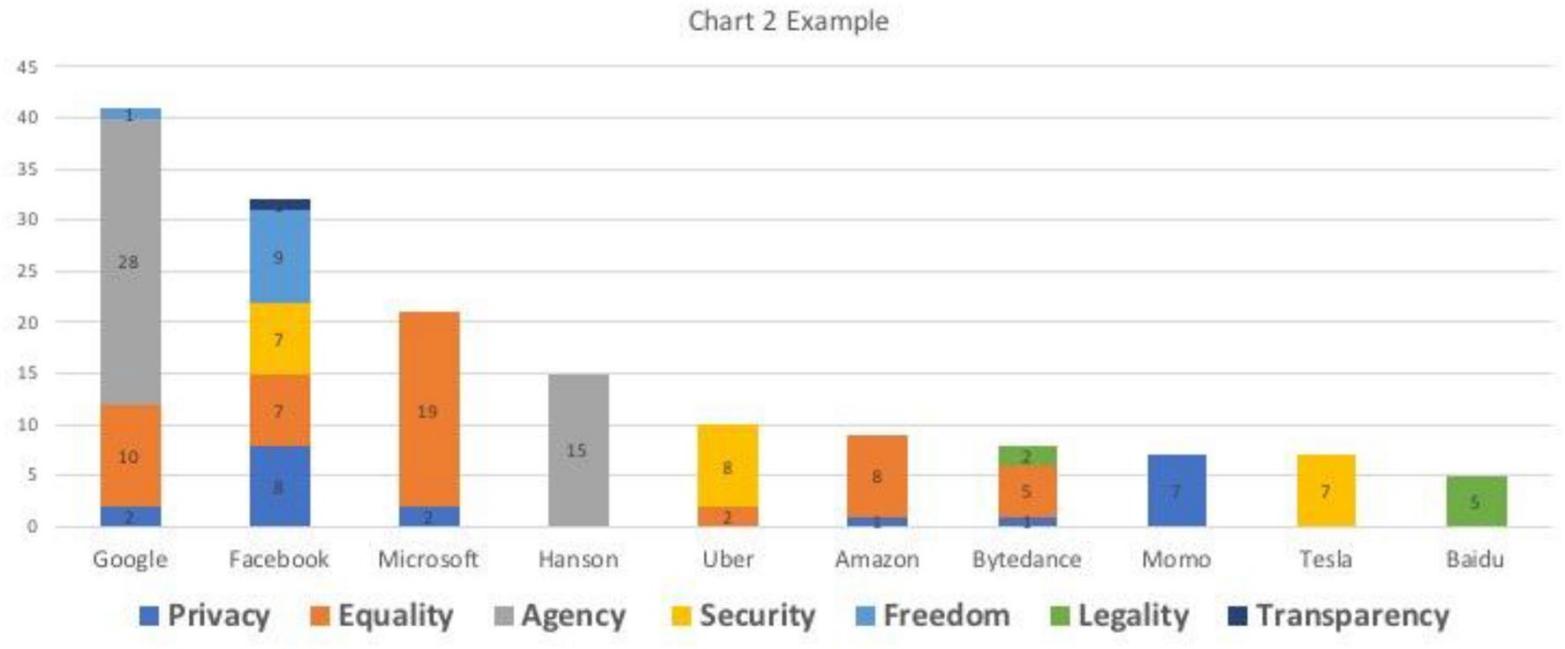
Example

**Chart 3:**
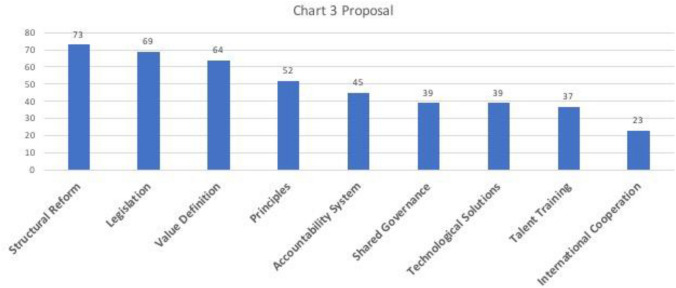
Proposal

**Chart 4:**
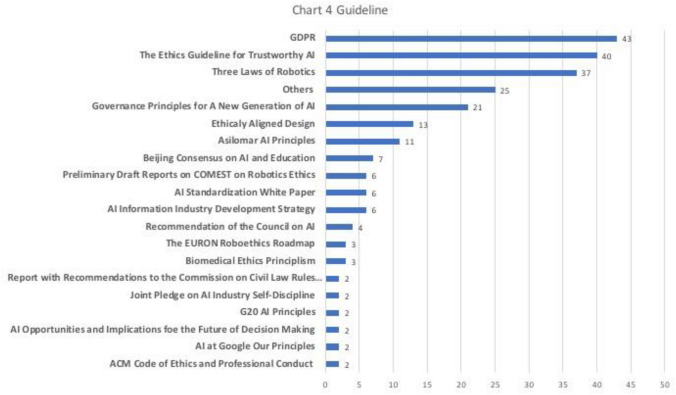
Guideline

**Chart 5:**
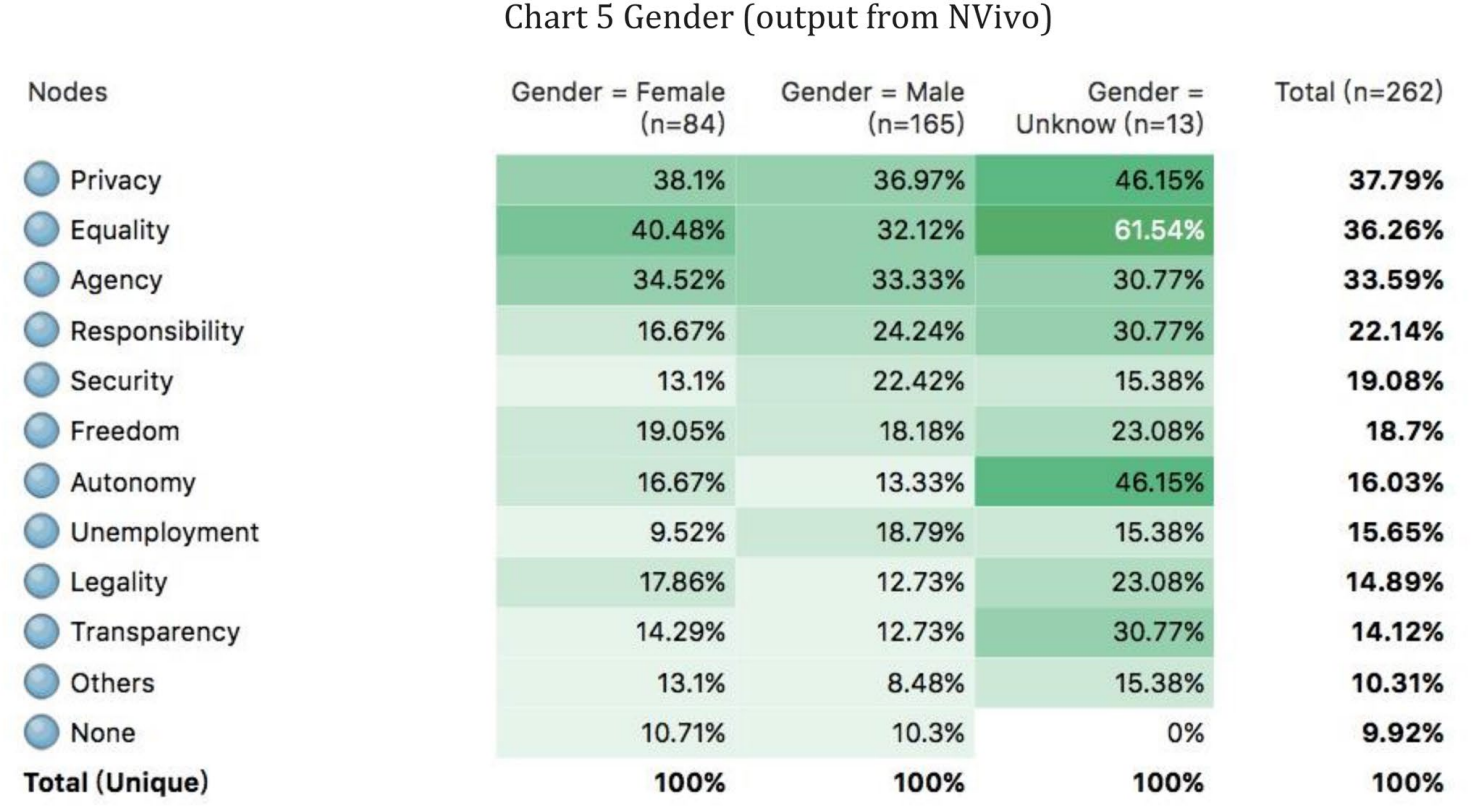
Gender (output from NVivo)

**Chart 6:**
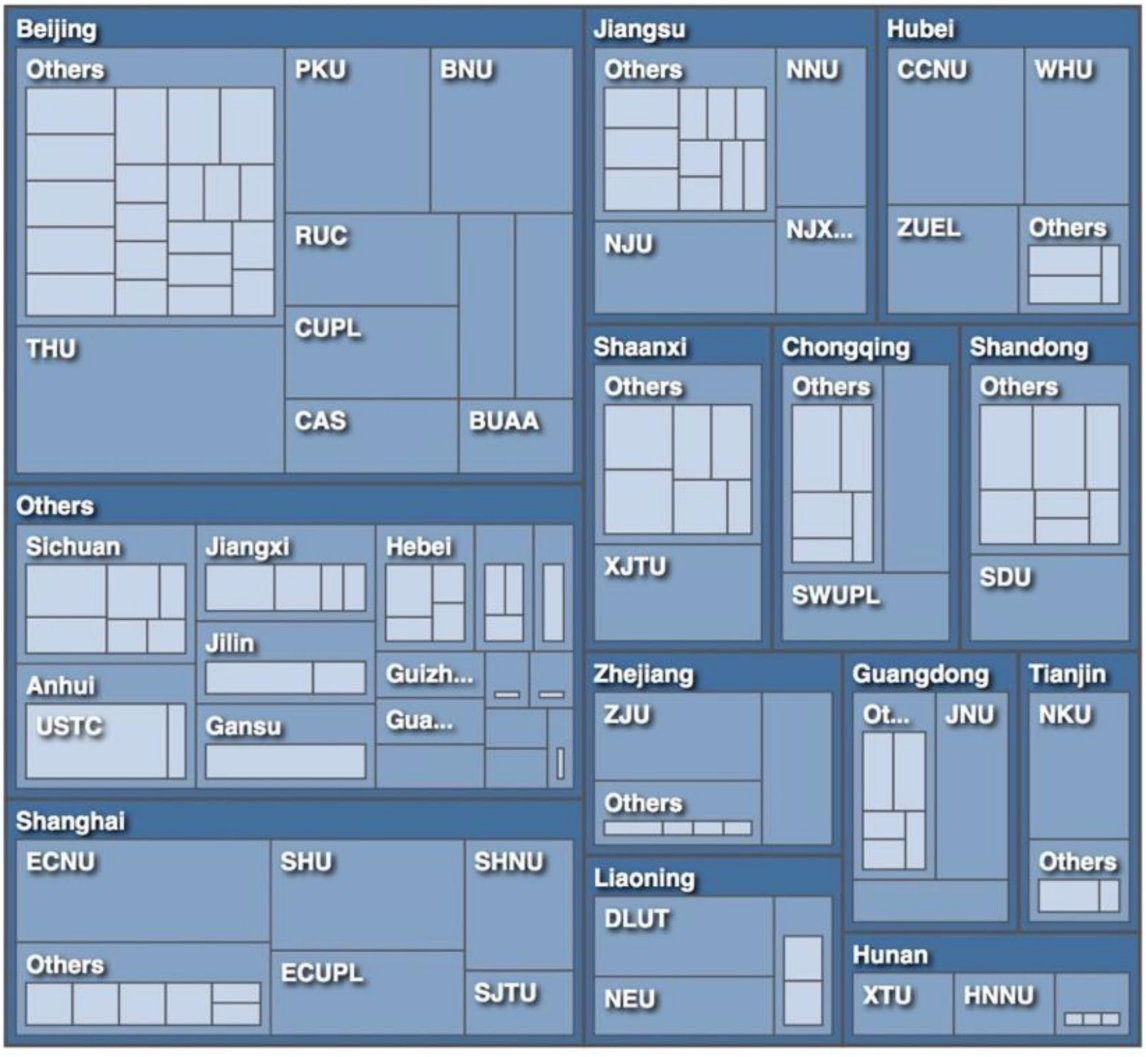
Affiliation (output from NVivo)

Most of the concerns from these prominent scholars have evolved into the aforementioned guidelines from BAAI and MOST, given their dominance of those committees. Apart from those universal principles, there are in fact some distinguishing concepts in these Chinese guidelines. In *Beijing AI Principles*, for example, there is a principle calling for *optimizing symbiosis* (优化共生) between human and AI, which stems from the traditional Chinese philosophy of harmony (和谐). As Zeng Yi, the leading author of *Beijing AI Principles*, explained in an interview,

“…many AI ethical principles are human-centered. …Such a design framework for the ethical principles of AI is somewhat short-sighted in my opinion. If it is human-centered, then the relations between humanity and the nature may not be well portrayed. …Besides, the future AI systems could become self-conscious or become a moral agent. If that’s the case, if we remain human-centered, our current depiction of AI ethical principles would be quite inappropriate. Current AI ethical principles is always about AI models and applications, and what criteria they should meet. However, I believe in a future society where humans and AI coexist, we should emphasize not only what kind of principles AI systems should try to uphold, but at the same time, in order to adapt to such a society, human beings also need to transform.”

Zeng Yi interviewed by Berggruen Institute 2019

As a member of UNESCO Ad Hoc Expert Group on Recommendation on the Ethics of Artificial Intelligence, Zeng Yi proposed to include this concept of harmonious symbiosis as a core value in the recommendation. Although the proposal was approved by several Asian and African ad hoc expert groups and government representatives, because some other representatives could not accept the terminology of “harmony” and “a community with a shared future for mankind (人类命运共同体)”, it was reframed to “living in peaceful, just and interconnected societies” in the final draft that was adopted by all 193 member states of UNESCO (Tian 2021). Even though the recommendation did not directly adopt the expression of “harmonious symbiosis”, relevant ideas were retained as Line 24 of the recommendation demands that the processes of the life cycle of AI systems should not threaten the coexistence between humans, other living beings and the natural environment (UNESCO 2021). This was considered by experts of various countries as a substantial embodiment of incorporating non-Western values into the global agreement on AI ethics (Tian 2021). It is evident that Chinese ways of thinking are more or less gaining its influence on those international negotiations and eventually the global standards. Therefore, understanding the Chinese discourse on AI ethics, as argued especially the academic discourse, becomes particularly important.

## Methodology

To offer an elaborate representation (instead of representation) of the Chinese academic discourse on AI ethics, this article employs a hybrid version of systematic review (Stahl et al. 2016), combining narrative review and statistical meta-analysis. The former relies on qualitative analysis to gain deeper understandings of particular issues and the latter quantitatively assess the discourse as a whole. The utilized methodology works indeed a lot like an AI algorithm, with selected journal articles being raw data, coding and categorizing process being algorithms, and findings being outputs. However, unlike most AI applications in the real world, the methodology here does not struggle with issues like algorithm black box. To consolidate the trustworthiness and reveal the inevitable bias of this article, the following sections describe the methods employed in selecting and sampling relevant literature, the process of coding and categorization, and the limitations.

### Selection of the literature

Since the focus of this article is on AI ethics in Chinese academia, the literature search was conducted in the database from which Chinese scholars and researchers would acquire their firsthand academic information, which is China National Knowledge Infrastructure (CNKI). Degree dissertations and conference papers were not targeted considering they would eventually evolve into journal articles. In addition, to ensure the quality of articles as well as avoid duplication, only CSSCI (Chinese Social Sciences Citation Index, which arguably is the most influential index in China) journals were chosen as the article source. Furthermore, since the third wave of AI started in the early 2010s, one decade of publications from 2011 to 2020 were included. Having decided on the type and source of publication and fixed the range of dates, CNKI was searched using “AI ethics /人工智能伦理” as the search term. The search was run on 1st of January 2021 and replicated on 23rd of March 2021. The results were both 328 hits, with 289 from 2020, 36 from 2019, and only 3 from previous publication years.^1^

All the result articles were collected and uploaded into NVivo 12 for sampling and coding. To ensure their relevance to the research, a three-step sampling method was employed. First, the title, abstract, and key words of each article were checked to assess whether it was obviously an academic publication focus on AI ethics. If not, it was then excluded. During this step, 55 articles were excluded due to irrelevance. Second, after starting systematic review on the remaining 273 articles, exclusion was continued through full analysis. Namely, the sampling was operated simultaneously with coding. Another 11 articles were excluded in the end of coding process. Lastly, in case of arbitrary decision, all the excluded articles were double-checked, and none was re-included. Altogether, 262 articles were sampled.

### Coding and categorizing

The scheme for coding and categorizing was rather straightforward. First, the target information was set. To answer the question of “what”, the first group of categories consists of discussed ethical issues as “*Issue*”,^2^ and exemplified AI scandals as “*Example*”; to answer the question of “how”, the second group consists of the recommended proposals for addressing the issues as “*Proposal*”, and the referred ethical guidelines as “*Guideline*”; finally, to answer the question of “who”, the third group consists of genders of both first and second authors as “*Gender*”, and their affiliations as “*Affiliation*”. Subsequently, those samples were read line by line to highlight the words, sentences, and paragraphs that fell into these categories, and those references were coded with their original wording. Noteworthily, some types of information were not available in several samples. In this case, those samples would have a code as “*None*” in the corresponding category. Besides, multiple entries in the same category were possible, if for instance more than one issue were discussed in the same sample. Consequently, each sample had at least one code in each category, namely at least six codes per sample. In total, there were 2847 codes (Issue: *n* = 745; Example: *n* = 416; Proposal: *n* = 585; Guideline: *n* = 344; Gender: *n* = 376; Affiliation, *n* = 381). Finally, codes within the same category were divided into several subcategories for further analysis. Information about subcategories and detailed analysis of each category can be found in Sect. 4.

### Limitations

Three major limitations are acknowledged in this article. First, this article collected only articles from the “official” Chinese academia. Yet private companies often invite influential scholars to publish on their own “semi-academic” channels, such as Tencent Research Institute and Alibaba DAMO Academy. Those articles were not collected, resulting in a short of cross-domain publications. Second, this article used “artificial intelligence ethics/人工智能伦理” as the only search term, which might have filtered the result by default. In the Chinese context, scholars might use “治理(governance)” instead of “伦理(ethics)” when addressing the ethical concerns of AI. Consequently, some governance-oriented articles might have been omitted. Third, the result hits under exactly same search term might vary significantly between dates due to algorithm or index updates, which was found to be a prevalent phenomenon in other databases (Bramer 2016). In fact, the search was replicated again on 3rd of July 2021, the result became 332 hits, with four more entries. When the same search was conducted in January 2022, there were much more new entries in the result. After received the inquiry, a staff member of CNKI explained the research results differed because “*journals’ editorial office may delay their article submission to our product team, or they asked to delay the time to launch to CNKI platform*”.^3^ It would be reasonable to believe that in order to improve their metrics, journals wanted to publish the articles they regarded as important in CNKI first, and those “less important” articles sometime later. Moreover, a radical reform of research assessment was recently launched in China to strengthen the local relevance of research, encouraging articles to be published in high-quality Chinese journals (Zhang and Sivertsen 2020). Therefore, it is expected the search result will have more entries. Therefore, instead of a systematic review of all the articles on AI ethics in Chinese academia, this article can only represent the discussions in the sampled 262 articles.

## Findings

### Issue

As shown in Chart 1, the most discussed issues about AI ethics in Chinese academia follow an order of “PEARSFAULT (Privacy, Equality, Agency, Responsibility, Security, Freedom, Autonomy, Unemployment, Legality, Transparency, and others)”. Privacy-related issues were the most discussed among 262 samples (*n* = 99), which contradicts to the opinion claiming privacy is not concerned in China (see for example, Jacobs 2018). The second most discussed subcategory of issue was equality (*n* = 95). This included bias and discrimination at the individual level. The most frequently mentioned problem was the “big data backstabbing/大数据杀熟”, which denotes the e-commerce platforms’ use of algorithms to profile customers and market the same product at different prices to different customers. However, other discriminations such as AI being sexist, ageist, and racist were rarely under explicit analysis but briefly mentioned as examples. Rather, Chinese scholars seemed to be more inclined to talk about inequalities from AI technologies at a broader societal level. For instance, many mentioned the phenomenon of enlarging digital divide, referring to the unequal access to AI technologies. Another major societal concern was AI helping tech companies to become monopolies, resulting in unfair allocation of benefits and further class stratification.

Unlike the previous two concrete problematics, the third most discussed issue was more abstract, concerning AI posing threats to human agency (*n* = 88). “*Can someone marry an AI? Should artificial general intelligence be considered as a moral agent? Or, what if AI singularity happens?*” Such questions were centered on the very fundamental human-centrism, elevating debates on ethics to a philosophical level. Some might regard those inquiries as alarmist, yet depending on the perspective from which scholars argue, they can be surprisingly relevant in the present times. From a perspective of responsibility allocation, those are far from being simply futuristic. Driverless vehicles causing traffic accident, AI journalists autogenerating fake news, and the aforementioned discriminations have all occurred and led to moral dilemmas where harm is made yet no one to blame. From a perspective of legality, clarifying the role of AI in legislation, especially in penal law and intellectual property law, is put on agenda by many governments. Responsibility and legality were, therefore, respectively the fourth (*n* = 58) and the ninth (*n* = 39) most concerned subcategories. In the fifth position was the concerns over security (*n* = 50), which was also multilayered. While most issues in this subcategory addressed technological malfunctions in the human–AI interactions, nearly half of the codes shared a theme of national security. Many Chinese scholars are concerned that AI might escalate the arm race and catalyze wars. Besides, AI technologies like deepfake can be used by terrorist groups or foreign hostile powers for fake news dissemination, political propaganda, and public opinion manipulation (Shi et al. 2020).

The sixth most concerned problems fell into the subcategory of freedom (*n* = 49). Interestingly, in the original wording, very few Chinese scholars used “freedom/自由” itself. Instead, they discussed those phenomena that implied the shrinking of some types of freedom, such as information cocoon (or filter bubble, denoting the shrinking access to different opinion), homogenization (meaning AI as teacher, judge, or any other kind of evaluators will limit the users’ freedom to learn, defense, or create). In fact, the potential personal freedom intrusion from AI surveillance was discussed in the name of privacy rather than *freedom*. What came after freedom was the concerns over autonomy (*n* = 42), which was raised in contexts where users became too dependent on AI applications to function as a normal human. For instance, older adults might reduce the real human-contact if they had an AI robot as companion. Lastly, many expressed concerns about AI raising the unemployment rate, which was the eighth most discussed issue (*n* = 41); as well as the problem of algorithm black box, which eroded the transparency and trustworthiness of AI (*n* = 37).

### Example

Only less than half of the samples referred cases of AI scandals happened in the reality to support their discussions (*n* = 120). The most frequently exemplified cases (*n* ≥ 5) were shown in Chart 2. Google (*n* = 41), Facebook (*n* = 32), Microsoft (*n* = 21), Hanson (*n* = 15), Uber (*n* = 10), and Amazon (*n* = 9) were the first six most exemplified institutions of unethical use of AI, which are all foreign tech companies. Local tech companies, on the other hand, appeared less frequently on the list, with entries of only Bytedance (*n* = 8), Momo^4^ (*n* = 7), and Baidu (*n* = 5). It seems that Chinese scholars concentrated their criticism on the foreign tech companies, instead of the local ones, even though there have been several domestic AI scandals such as the death of Wei Zexi (Heng 2016), the dystopian Focus1 (Feng 2019), and the first legal case over facial recognition (Ye 2020). Further, there was very limited discussion on the AI applications that were initiated by the government, such as facial recognition systems for surveillance and social credit systems. In terms of types of concerns, while most institutions were listed for only one or two, Facebook was involved in five subcategories, including privacy, equality, security, freedom, and transparency. Regarding the most exemplified individual cases, Google’s AlphaGo (*n* = 28) that caused concerns over AI threatening human agency ranked the first position. Microsoft’s Tay (*n* = 19) was criticized for its discriminative responses, occupying the second, and Hanson’s Sophia (*n* = 15) came to the third with its controversial Saudi Arabian citizenship. The popularity of these three cases is fairly understandable, given their association with Sci-Fi and massive media dissemination. Other cases in which AI played a substantial yet invisible role received less attention, such as fatal accident caused by an Uber’s self-driving vehicle (*n* = 8) and the Facebook-Cambridge Analytica scandal (*n* = 7).

### Proposal

The majority of the samples raised proposals to address the ethical concerns (*n* = 166). Each sample gave on average two to three proposals. As shown in Chart 3, those proposals can be further divided into nine subcategories, namely structural reform (*n* = 73), legislation (*n* = 69), value definition (*n* = 64), ethical principles (*n* = 52), accountability system (*n* = 45), shared governance (*n* = 39), technology solutions (*n* = 39), talent training (*n* = 37), and international cooperation (*n* = 23). Structural reform became the first because it covered the establishment of regulatory processes or institutions in the all life circle of an AI application, including ethics review committee and algorithm auditing in the design stage, penalties for the wrong use of AI, and incentives like subsidies for approved effective and responsible applications. Legislation, or to put it more simply, issuing new laws on AI was the second most popular proposal. This was considerably more advocated than another proposal, issuing ethical principles (the fourth), which implied the Chinese scholars would prefer to ask the government to draft strong-binding laws than urge the institutions to issue their own weak principles. If the academic discourses indeed reflect policy changes, it is expected that there will be more concrete laws governing the use of AI in China. Value definition, as the third most popular proposal, referred to the standard-making of ethical considerations about AI. One concept that Chinese scholars frequently mentioned was the dichotomy of *zweckrational–wertrational*. The former denoted acting based on the rational evaluation of consequences (for example, consequentialism) while the latter was characterized by striving for (usually irrational) reasons or motives intrinsic to the actor (for example, deontology). In fact, most scholars proposing value (re)definition argued that the recent AI development had been driven by an excessive zweckrational thus causing ethical problems. Nonetheless how to balance two value standards in practice and who should be the actor were not clearly pointed out.

Occupying the fifth position, accountability system represented those proposals to clarify the responsibility of different stakeholders involved in the design, use, and governance of AI. In particular, the majority of proposers had an urge to hold AI companies accountable for more responsibility. Shared governance, which represented those proposals to include multiple stakeholders, especially the public, in the governance of AI, was the sixth. There were same number of scholars proposing technological solutions to address the ethical issues. For instance, blockchain was believed to be able to record the manufacturing and development progress of AI, so regulators can keep tracking, evaluating, and eventually preventing the potential unethical uses (Cai 2020). Lastly, there were another two subcategories, talent training and international cooperation. The former contained those proposals to embed the ethics education into the AI talent training system, those to familiarize the general public users with the rationale of AI, and all those to use education to make a difference. The latter, as the name suggested, called for more international cooperation on the governance of AI.

### Guideline

42% of the samples (*n* = 110) referred ethical guidelines to discuss ethical concerns of AI. Guidelines that appeared at least twice were listed in Chart 4 in their original names, while those appeared once were included in Others (*n* = 25). Among those samples, more than half referred guidelines from EU including GDPR (*n* = 43), *Ethics Guidelines for Trustworthy AI* (*n* = 40), and *EURON Roboethics Roadmap* (*n* = 3). Besides, 37% referred guidelines were from academia (*n* = 41), 26% referred guidelines were from Chinese government (*n* = 28), and 16% referred were from international organizations (*n* = 17). The most referred guidelines from academia, Chinese government, international organizations were, respectively, *Isaac Asimov’s Three Laws of Robotics* (*n* = 37), *Governance Principles for A New Generation of AI: Develop Responsible AI* (*n* = 21), and *UNESCO’s Beijing Consensus on AI and Education* (*n* = 7). This finding showed that Chinese scholars discuss the European guidelines much more often than the ones from the USA. In addition, in terms of the domestic guidelines, Chinese scholars are more familiar with *Governance Principles for A New Generation of AI: Develop Responsible AI*, which was a full initiative from the government, rather than BAAI’s *Beijing AI Principles*, or AIIA’s *Joint Pledge on Self-discipline in the AI Industry*, which were both issued by the private-sector with governmental endorsement. Mostly, those guidelines were referred as either the ethical standards one should follow or a good example of AI governance. Nonetheless, there were also scholars analyzing those guidelines in the bigger contexts. For instance, *Ethics Guidelines for Trustworthy AI* was regarded as the EU’s strategic consideration of enhancing its normative power and striving for the right to make international standards (Yin and Fang 2020).

### Gender

Among all the first and second authors, male authors accounted for 56% (*n* = 211), female authors accounted for 34% (*n* = 126), and unidentifiable authors accounted for 10% (*n* = 39). However, if counting only the first author, the share of males rose up to 63% (*n* = 165) and the share of females decreased to 32% (*n* = 84). Male dominance was found to be apparent in the discourse on AI ethics in Chinese academia, which was similar to the finding from Thilo Hagendorff. In fact, the gender distribution of the scholars who drafted the aforementioned three Chinese ethical guidelines was even more unbalanced. There were no female members in those committees at all. Nonetheless, this study did not find strong evidence proving that females have different moral reasonings than males. Thilo Hagendorff agreed with Carol Gilligan that men address moral problems through logic-oriented and rational “ethics of justice” while women interpreting them within an emotion-oriented and empathic “ethics of care” framework (Gilligan 1982). He, therefore, argued that male dominance was the reason why transparency, justice and fairness, responsibility, and privacy are the most frequently mentioned principles while AI in contexts of care, help, welfare, or ecological networks are barely mentioned (Hagendorff 2020). However, this is not necessarily the case in Chinese academia. As shown in Chart 5, this study did not find significant differences between the prioritized ethical issues of female and male authors. Both shared similar patterns and concerned about privacy, equality, and agency the most. Therefore, one can argue that even there is a more balanced proportion of female authors writing those ethical guidelines, guidelines about welfare or ecological networks may remain omitted. After all, Gilligan’s ethics of care framework that argues women approach ethics differently from men was raised in the 1980s and has already received many critiques from other feminist scholars in the 1990s (Senchuk, 1990). Besides, the female moral reasoning may have changed since 1980s.

### Affiliation

The authors of 262 samples were from 198 different institutions all over China. Each provincial level area had at least one institution on the list. The vast majority of the institutions were public universities or colleges and only nine were private. As shown in Chart 6, 63% of the samples were produced in institutions located in eastern China (*n* = 164), with 15% from central China (*n* = 40), 17% from western China (*n* = 44), and 5% from northeastern China (*n* = 14). The most published institutions were Tsinghua University (THU, *n* = 15), East China Normal University (ECNU, *n* = 11), Peking University (PKU, *n* = 9), Beijing Normal University (BNU, *n* = 9), and Shanghai University (SHU, *n* = 9), which were all elite universities located in the east. These findings suggested a prevailing research interest in AI ethics in all parts of China, yet the current discourse is dominated by the prestigious universities from eastern China, particularly Beijing and Shanghai. It seems that no matter domestically in China or internationally, discourse on AI ethics is always shaped by the more economically developed regions.

## Discussion

Overall, this article found the discussions about AI ethics in Chinese academia to be highly diverse, similar to the finding in online public discourse from Mao and Shi-Kupfer (2021). Sessions 4.1 and 4.2 offered forthright answers to the question of “what”, concerning the most discussed ethical issues of AI in Chinese academia and those being left out. Among the diverse Chinese literature on AI ethics, one group of scholars expressed their short-term concerns on how to regulate AI algorithms, models, and applications, to make them abide by the existing ethical principles. The ethical concerns from those scholars were centered on the globally convergent principles, which was well reflected in the popularity of concerns over privacy, equality, and responsibility. In other words, although the prescribed methodologies may differ from those proposed in the West (which is discussed below), this group of Chinese scholars saw the similar problems, including privacy intrusion (Lin and Chen 2020), algorithm discrimination (Wang 2020), and the problem of responsibility allocation (Pan and Yang 2020). Besides, not only the most concerned issues, but the lack of mentioning about sustainability- and solidarity-related issues is also a similarity shared by the Chinese academic discourse and the international guidelines.

On the other hand, another group of scholars focused their debates on the long-term concerns about the more futuristic version of AI, be it artificial general or super intelligence. This was reflected by the fact that agency came only after privacy and equality as the third most concerned issue. Similar to Zeng Yi, those scholars saw AI as a potential moral agent and discussed how they might change the current human society and how we should prepare for that future. Interestingly, this article found fundamentally contrasting arguments within this group. One school held onto the fundamental principle of human-centrism. Yet this principle was questioned by another school of Chinese scholars who mostly shared a stance that the eastern cultural heritage will entail a different view on human–AI relations than the West. For instance, Song Bing argued that the traditional Chinese three teachings, namely Confucianism, Buddhism, and Taoism, all share a non-humancentric moral root, which explained why the Chinese were less suspicious and fearful of AI as a moral agent than those in the West, resulting in a more accepting environment for AI (Song 2020). This cultural relativist view can be found in English literature as well (for example Gal 2020; Fung and Etienne 2021). However, in terms of public perception of AI as moral agent, Mao and Shi-Kupfer (2021) found different results from Zeng et al. (2020). The former found that many Chinese netizens were concerning the humanity in the long run, while the latter found the online opinion to be mostly featured with discussions about the economic potential of the technology with little critical debate. This contrast might be caused by their searching on different platforms, or the fact that discussions about AI ethics have just boomed in recently years when AI scandals skyrocketed. Either way, whether (if yes, then why) Chinese people have a more positive perception of AI, particularly strong AI, is yet to be examined in a more cultural context. In addition, this article noticed that AI scandals Chinese scholars discussed were mostly foreign cases. Critical analysis on the domestic cases were less carried out, which is considered rather intriguing. Why Chinese scholars have a tendency to not talk about the local cases? One possibility is that they are covering up the domestic slips, in order to avoid controversy and promote “positive energy”. As Lao Dongyan, a professor at School of Law Tsinghua University, wrote in her later censored article, “…*today, no matter the public or the government, including the younger generation, they do not welcome so much the intellectuals who criticize the problems in the society…*” (Lao 2022) Or perhaps it was the journals that filtered out those pieces that criticized domestic slips? While explaining this phenomenon is another important and interesting topic, it will require different research plan and methods that are beyond the scope of this article.

In terms of the solutions proposed and preferred by Chinese scholars, Sessions 4.3 and 4.4 provided some valuable insights. In Chinese academia, there was a shortage of proposals for conversation and cooperation mechanisms, which is particularly worrying under the current tensions between China and the USA. Rather, Chinese scholars raised a wide range of solution proposals that were targeting, respectively, different domestic stakeholders including the government, the tech companies, and also the general public. It was evident that Chinese scholars preferred strong-binding regulations than those weak ethical guidelines. In fact, the newly enacted *Personal Information Protection Law* (PIPL), which bears a resemblance to the European *General Data Protection Regulation* (GDPR), has filled the lack of a comprehensive regulatory framework for personal data protection in China (National People’s Congress 2020). However, it remains yet to be seen how the new law will be implemented in terms of assessment and penalties and how interoperable it is with GDPR. More recently, to stop the manipulative uses of algorithm, China approved the first of its kind specific regulation on algorithm (Cyberspace Administration of China 2022). If China’s attempts to rein in algorithms prove successful, they could in fact imbue these approaches with a kind of technological and regulatory soft power that shapes AI governance regimes around the world (Sheehan 2022).

The birth of such strong-binding regulations certainly echoed the calls from academia and they are expected to reduce the misuse of AI systems, yet can we conclude that in China academic voices about AI are indicative of the local policy changes? Or is it merely a coincidence as the government is tightening grip on those tech giants? To put it differently, what is the essence of such policy changes in China, a response to the citizens’ need, a further centralization of power, or perhaps a mixture of both? Answers to such questions are crucial for understanding the Chinese policies on AI ethics, yet finding them requires more nuanced analysis on the domestic socio-political contexts, in particular the interactions among different stakeholders in the AI ecosystem. Since academia, as argued in this article, is playing a role of increasingly importance, it is certainly worthy to keep observing closely how Chinese scholars will continue to participate in and shape the AI governance in China.

Sessions 4.5 and 4.6 mapped out whose voices were dominating and whose were in the marginal. Findings suggest that the Chinese academic discourse on AI ethics was dominated by male authors and those from elite universities located in the wealthier eastern China, which was similar to the landscape of international discourse (Jobin et al. 2019; Hagendorff 2020). However, unlike the previous studies (Hagendorff 2020), this systematic review did not find significant differences of the ethical concerns about AI between female and male scholars. Privacy, equality, and agency were the most concerned issues for both groups. Rather than on gender, ethical concerns over AI may highly rely on the disciplinary backgrounds of the authors. In fact, only seven out of 262 samples had an author from hard sciences background and all other authors were from a social sciences or philosophy background. Thus, the voices of computer scientists, engineers, and mathematicians were missing from the current discourse on AI ethics in China.

Climate change and the covid-19 pandemic have reminded us that we inhabit the same planet and we are bound by the same fundamental laws of universe. While some Chinese characteristics do exist in the way how they see AI as a potential moral agent, this article found that Chinese scholars share predominantly same concerns with their international counterparts over AI algorithms, models, and applications, indicating a significant common ground for cross-nation and cross-culture cooperation on the governance of AI as a disruptive technology.

## Supplementary Information

Below is the link to the electronic supplementary material.

Supplementary file1 (DOCX 32 KB)

## Data Availability

All sample articles are available from www.cnki.net/, a list of coded articles is attached as supplementary document.

## Footnotes

1 A list of collected articles in the chronological order can be found in supplementary document.

2 Coded only when at least one paragraph of discussion on the raised issue were provided in the text.

3 The author first contacted Inga-Lill M. Blomkvist, the librarian of Nordic Institute of Asian Studies from which the author got the access to CNKI publications. She forwarded the inquiry to the staff member of CNKI and later kindly sent back the answer, which the author is very much grateful for.

4 Momo is a Beijing Internet company established in 2011, known for the homonymic social search and instant messaging mobile app that is considered to an equivalent to Tinder.
