# Supplementary material for: AI ethics with Chinese characteristics? Concerns and preferred solutions in Chinese academia
Source: AI Soc. 2022 Oct 17:1–14. Online ahead of print. doi: 10.1007/s00146-022-01578-w (PMC9574803; doi:10.1007/s00146-022-01578-w)
Supplement: Supplementary file 1 — Supplementary file1 (DOCX 32 KB) [file 146_2022_1578_MOESM1_ESM.docx]

**Supplementary document: List of Collected Articles**

*Texts in strikethrough represent the excluded article.

| No. | Time | Author(s) |  | No. | Time | Author(s) |  | No. | Time | Author(s) |
| --- | --- | --- | --- | --- | --- | --- | --- | --- | --- | --- |
| 001 | 20130510 | Wang S.Y.& Zhao J. |  | ~~043~~ | ~~20200106~~ | ~~Gretzel U.& Xiang Z.~~ |  | ~~085~~ | ~~20200225~~ | ~~Guo W.~~ |
| 002 | 20130615 | Wang S.Y.& Cui W.Q. |  | 044 | 20200106 | Wang W.J. et al. |  | 086 | 20200226 | Xu Y.& Huang F.S. |
| 003 | 20140110 | Wang D.H. |  | 045 | 20200109 | Zhang A.J. |  | 087 | 20200226 | Yu G.M.& Geng X.M. |
| 004 | 20190311 | Xiong B. |  | 046 | 20200110 | Chen W.H. |  | 088 | 20200226 | Peng L. |
| 005 | 20190912 | Chen F.& Xu.X. |  | 047 | 20200110 | Cui W.Y. |  | 089 | 20200226 | Xue F.& He Y.F. |
| ~~006~~ | ~~20190917~~ | ~~Chen T.& Han Q.~~ |  | 048 | 20200110 | Miao M.H. |  | 090 | 20200228 | Gu J.F. et al. |
| 007 | 20190919 | Yan K.R. |  | 049 | 20200113 | Zhu J.J. |  | 091 | 20200303 | Zhang F.F. |
| ~~008~~ | ~~20191010~~ | ~~Introduction~~ |  | 050 | 20200115 | Wang T.E. |  | 092 | 20200304 | Cai L.Y.& Gong Z.W. |
| 009 | 20191010 | Shen Y.& Wang Q. |  | 051 | 20200115 | Cao X.M.& Xian C.X. |  | 093 | 20200305 | Wu T.L. |
| ~~010~~ | ~~20191018~~ | ~~Wang H.Y.& Long Y.~~ |  | 052 | 20200115 | Xu H.L. |  | 094 | 20200305 | Zhang B. |
| 011 | 20191031 | Cheng P. |  | 053 | 20200115 | Long W.Q. |  | 095 | 20200306 | Xiang Y.H.& Wang X.H. |
| ~~012~~ | ~~20191110~~ | ~~Jin D.N.~~ |  | ~~054~~ | ~~20200115~~ | ~~Zhang D.& Cui G.Z.~~ |  | 096 | 20200310 | Pan E.R.& Yang J.F. |
| 013 | 20191113 | Cui Z.L. et al. |  | 055 | 20200115 | Dai Y.B. |  | 097 | 20200310 | Pan B. |
| ~~014~~ | ~~20191115~~ | ~~Deng D.Q.~~ |  | 056 | 20200115 | Wang Y.L.& Deng G.M. |  | 098 | 20200310 | Zhang J.M.& Xu Y.L. |
| 015 | 20191115 | Liu F.X. |  | 057 | 20200115 | Geng X.M.& Yu G.M. |  | 099 | 20200310 | Sun S.G. |
| 016 | 20191115 | Li Z.T.& Luo Y. |  | 058 | 20200115 | Lin A.J.& Liu Y.H. |  | 100 | 20200310 | Zhao J.& Li W.Z. |
| ~~017~~ | ~~20191115~~ | ~~Sun X.& Wu H.L.~~ |  | 059 | 20200117 | Que T.S.& Zhang J.T. |  | 101 | 20200311 | Zhang Y.B. |
| 018 | 20191115 | Bie J.H. |  | 060 | 20200117 | Chen S. |  | 102 | 20200313 | Sun H.Y. |
| 019 | 20191115 | Zhang X.S. |  | 061 | 20200117 | Huang K.F.& Cai D. |  | 103 | 20200313 | Xie X.S. |
| ~~020~~ | ~~20191120~~ | ~~Zhao B.~~ |  | 062 | 20200118 | Wang L. |  | 104 | 20200315 | Liu X. |
| 021 | 20191122 | Chen S.S. |  | ~~063~~ | ~~20200119~~ | ~~Jiang F.G. et al.~~ |  | 105 | 20200315 | Wang L.B. |
| 022 | 20191122 | Yan J.H.& Wang Z.H. |  | ~~064~~ | ~~20200120~~ | ~~Li F.H. et al.~~ |  | 106 | 20200315 | Xue B.Q. |
| 023 | 20191127 | Zou D.D. |  | ~~065~~ | ~~20200120~~ | ~~Yu Y.F.& Li Y.~~ |  | ~~107~~ | ~~20200315~~ | ~~Hao Q. et al.~~ |
| 024 | 20191205 | Tan W.Z. |  | ~~066~~ | ~~20200121~~ | ~~Lei L.~~ |  | 108 | 20200320 | Cai Z.H.& Zhao Y.H. |
| ~~025~~ | ~~20191205~~ | ~~Zhang Y.& Wang Y.W.~~ |  | 067 | 20200205 | Fu Y. |  | 109 | 20200320 | Liao S. |
| 026 | 20191209 | Zhang W. |  | 068 | 20200205 | Chen Y. |  | 110 | 20200320 | Zhou Q.& Wen X.Y. |
| 027 | 20191215 | Xu W. |  | 069 | 20200210 | Zhang L.Y. |  | 111 | 20200320 | Li Q. et al. |
| 028 | 20191215 | Zhao R.Q. |  | 070 | 20200210 | Xu W. |  | ~~112~~ | ~~20200320~~ | ~~Liu C.C. et al.~~ |
| 029 | 20191218 | Wang Y.Q. |  | ~~071~~ | ~~20200210~~ | ~~Liu Y.& Hu Q.X.~~ |  | 113 | 20200320 | Feng C.Y.& Chen X.Y. |
| ~~030~~ | ~~20191220~~ | ~~Mei L. et al.~~ |  | ~~072~~ | ~~20200210~~ | ~~Yuan L.P.& Chen C.N.~~ |  | 114 | 20200320 | Li N.& Chen J. |
| ~~031~~ | ~~20191220~~ | ~~Zhang J.~~ |  | ~~073~~ | ~~20200210~~ | ~~Wu J.X.~~ |  | ~~115~~ | ~~20200320~~ | ~~Yuan L.P.& Chen C.N.~~ |
| 032 | 20191220 | Wang C. |  | 074 | 20200210 | Yang Q.F. |  | 116 | 20200320 | Wang W.Q. et al. |
| 033 | 20191225 | Fu C.Z. |  | 075 | 20200210 | Chen X.P. |  | 117 | 20200320 | Li W.& Li J. |
| 034 | 20191225 | Yang T.J. |  | 076 | 20200210 | Zhao T.Y. |  | 118 | 20200320 | Feng X.Q.& Pan B.H. |
| ~~035~~ | ~~20191225~~ | ~~Discussion~~ |  | 077 | 20200215 | Wang J.Y.& Lu Z.X. |  | 119 | 20200323 | Yin J.Z.& Fang L.X. |
| 036 | 20191225 | Zhang A.J.& Li Y. |  | 078 | 20200215 | Zhao B.J. |  | 120 | 20200330 | Zhang S.Q. |
| 037 | 20191229 | Long W.Q. |  | 079 | 20200215 | Song J.J. |  | 121 | 20200401 | Ma X.D. |
| ~~038~~ | ~~20191230~~ | ~~Fan C.L.~~ |  | 080 | 20200217 | Hu Y.C.& Li Y.Y. |  | 122 | 20200401 | Lu X. et al. |
| ~~039~~ | ~~20191231~~ | ~~Shen Y. et al.~~ |  | 081 | 20200218 | Jiang Y.L.& Sun J. |  | 123 | 20200401 | Zhang X.J.& Dong X.H. |
| 040 | 20200103 | Li M.L. |  | 082 | 20200219 | Cheng H.D. et al. |  | 124 | 20200405 | Wang H.J.& Ru X.H. |
| 041 | 20200105 | Xu P.& Xu X.Y. |  | 083 | 20200220 | Guo X.L. |  | 125 | 20200405 | Chen C.F.& Xu F.Y. |
| 042 | 20200105 | Shuai Y.N. |  | 084 | 20200220 | Li M.D. et al. |  | ~~126~~ | ~~20200405~~ | ~~Speech~~ |
| No. | Time | Author(s) |  | No. | Time | Author(s) |  | No. | Time | Author(s) |
| 127 | 20200405 | Huang S.Z. |  | 169 | 20200520 | Yu Y.Z.& Hu F.G. |  | 211 | 20200714 | Sun W.P.& Fu Z.Q. |
| 128 | 20200405 | Sun D.C. |  | ~~170~~ | ~~20200520~~ | ~~Yan K.R.~~ |  | 212 | 20200715 | Wang K.Y.& Zhang G.D. |
| 129 | 20200407 | Zhang H.Z. et al. |  | 171 | 20200520 | Du P. et al. |  | 213 | 20200715 | Wang T.E. |
| 130 | 20200408 | Liu J. et al. |  | 172 | 20200520 | Zhao W.L. et al. |  | 214 | 20200715 | Liu L.& Yang X.L. |
| 131 | 20200409 | Mao G.J. |  | 173 | 20200527 | Guo H.N.& He Q. |  | 215 | 20200715 | Yang C.M.& Wei Q. |
| 132 | 20200410 | Yan K.R. |  | 174 | 20200528 | Peng H.M.& Liu Z. |  | 216 | 20200715 | Zeng Z.H. et al. |
| 133 | 20200410 | Du Y.Y. |  | 175 | 20200529 | Liu L.& Liu R. |  | 217 | 20200715 | Li Y.X. |
| ~~134~~ | ~~20200410~~ | ~~Wang Z.Q.~~ |  | 176 | 20200529 | Huang K.S. |  | 218 | 20200716 | Zhao Y. et al. |
| 135 | 20200410 | Xiao R. et al. |  | 177 | 20200529 | Guo W.M. |  | 219 | 20200716 | Kong X.W. |
| 136 | 20200410 | Ma H.X.& Zhu D.Q. |  | 178 | 20200601 | Di L.Y. |  | 220 | 20200718 | Zhang W.X. |
| 137 | 20200410 | He Z.F. |  | 179 | 20200601 | Wan K.& Ren Y.Q. |  | 221 | 20200720 | Wang Y.H. |
| 138 | 20200410 | Li X. |  | 180 | 20200601 | Deng G.M.& Li M. |  | 222 | 20200720 | Zhang W.G.& Wu Y.X. |
| 139 | 20200415 | Yang M.L. |  | ~~181~~ | ~~20200601~~ | ~~Zhan Z.H.& Zhong B.C.~~ |  | 223 | 20200725 | Huang M.F. |
| 140 | 20200415 | Wu H.J. et al. |  | 182 | 20200604 | Long W.Q. |  | ~~224~~ | ~~20200725~~ | ~~Zhong H.& Ma X.F.~~ |
| 141 | 20200415 | Ji W.M. |  | 183 | 20200604 | Wei D. |  | 225 | 20200727 | Chen G. |
| ~~142~~ | ~~20200415~~ | ~~Liu H.Y.~~ |  | 184 | 20200605 | Liu T.& Liu Q.X. |  | 226 | 20200728 | Zhang A.Y. |
| 143 | 20200415 | Luo Y.H. |  | ~~185~~ | ~~20200605~~ | ~~Chen P.~~ |  | ~~227~~ | ~~20200728~~ | ~~Lin J.& Zheng L.N.~~ |
| 144 | 20200417 | Li X.Y.& Zhang Y.Q. |  | ~~186~~ | ~~20200608~~ | ~~Wang G.W.~~ |  | ~~228~~ | ~~20200730~~ | ~~Zhang W. et al.~~ |
| ~~145~~ | ~~20200420~~ | ~~Fang B.& Hu R.D.~~ |  | 187 | 20200608 | Chen B. |  | 229 | 20200803 | Wang F.& Guo L. |
| 146 | 20200420 | Anwer Z.& Yao Y. |  | 188 | 20200609 | Lu H.Y. |  | 230 | 20200805 | Chen R.& Sun Q.C. |
| 147 | 20200420 | Ye X.D. |  | ~~189~~ | ~~20200610~~ | ~~Li X.M.~~ |  | 231 | 20200805 | Chen C.F.& Zhang M. |
| 148 | 20200420 | Liu X.C.& Li C. |  | 190 | 20200610 | Wu G.C. |  | 232 | 20200805 | He Z. |
| ~~149~~ | ~~20200420~~ | ~~Wang Z.X.& Song P.~~ |  | 191 | 20200610 | Li H.X.& Tian L. |  | ~~233~~ | ~~20200810~~ | ~~Zhao L.L. et al.~~ |
| 150 | 20200421 | Ma S.B.& Xu Y. |  | 192 | 20200615 | Zheng X. |  | 234 | 20200811 | Xiao S.S. |
| 151 | 20200425 | Yang Q.F. |  | 193 | 20200615 | Lu K. et al. |  | 235 | 20200811 | Li R.X. |
| 152 | 20200501 | Tian X.P. |  | ~~194~~ | ~~20200615~~ | ~~Zhao B.J.& Sun W.P.~~ |  | 236 | 20200813 | Tang D.X. |
| ~~153~~ | ~~20200505~~ | ~~Que T.S.& Zhang J.T.~~ |  | ~~195~~ | ~~20200615~~ | ~~Zhao X.M.& Li L.~~ |  | ~~237~~ | ~~20200815~~ | ~~Li H.Y.~~ |
| 154 | 20200505 | Zhu C. |  | 196 | 20200618 | Jia L. |  | ~~238~~ | ~~20200816~~ | ~~Zhang L.H.& Xiao K.~~ |
| 155 | 20200506 | Cai H.J. |  | 197 | 20200619 | Xu J.& Ma X.Y. |  | 239 | 20200820 | Sun L. |
| 156 | 20200510 | Wang H.J. |  | 198 | 20200620 | Cui Y.D. |  | ~~240~~ | ~~20200820~~ | ~~Gu M.Y.& Feng Z.Y.~~ |
| ~~157~~ | ~~20200510~~ | ~~Zhang J.Y.~~ |  | 199 | 20200620 | Xu F.L. |  | ~~241~~ | ~~20200821~~ | ~~Gao Q.Q.~~ |
| 158 | 20200510 | Shi J. et al. |  | 200 | 20200623 | Wu X.J. |  | 242 | 20200825 | Wang T.E. |
| 159 | 20200510 | Shang B.W. |  | 201 | 20200625 | Yu D.H. |  | 243 | 20200827 | Du L.Q.& Cheng J.X. |
| 160 | 20200510 | Zuo H.X.& Wang M. |  | 202 | 20200625 | Yu X.& Li L. |  | 244 | 20200901 | Sun T.L.Z. |
| 161 | 20200510 | Li B.C. |  | 203 | 20200625 | Zhou M. |  | 245 | 20200901 | Zhu H.L.& Tang C.M. |
| 162 | 20200512 | Deng Z.G. |  | 204 | 20200701 | Lv X.J. et al. |  | 246 | 20200909 | Zhang L.H. et al. |
| 163 | 20200515 | Wang S.Q. |  | 205 | 20200703 | Chen G. |  | 247 | 20200910 | He Q. et al. |
| 164 | 20200515 | Wang Y.H.& Wang Z. |  | 206 | 20200705 | Zhang J.W. |  | 248 | 20200910 | Wang Z.Y. |
| 165 | 20200518 | Sun B.& Zhou X.J. |  | 207 | 20200705 | Chen H.F. |  | 249 | 20200910 | Lan J. |
| ~~166~~ | ~~20200520~~ | ~~Jin H. et al.~~ |  | 208 | 20200708 | Liu W.B.& Liu J |  | 250 | 20200910 | Huang W.W. |
| ~~167~~ | ~~20200520~~ | ~~Yu Y.& Xie S.X.~~ |  | 209 | 20200710 | Wang D.& Zhang Z. |  | ~~251~~ | ~~20200910~~ | ~~Zhang Z.X. et al.~~ |
| 168 | 20200520 | Feng R. et al. |  | 210 | 20200710 | Li C. |  | 252 | 20200910 | Chen F.& Xu X. |
| No. | Time | Author(s) |  | No. | Time | Author(s) |  | No. | Time | Author(s) |
| 253 | 20200910 | Yan K.R. |  | 279 | 20201010 | Zhao Z.X.& Du X.X. |  | 305 | 20201120 | He M.S. |
| 254 | 20200915 | Jian X.X. |  | 280 | 20201015 | Dai Y.B. |  | 306 | 20201120 | Lu D. et al. |
| ~~255~~ | ~~20200915~~ | ~~Xu H.J.~~ |  | 281 | 20201015 | Tian Y.Y.& Han X.T. |  | ~~307~~ | ~~20201120~~ | ~~Xu Y.F. et al.~~ |
| ~~256~~ | ~~20200915~~ | ~~He Y.J. et al.~~ |  | 282 | 20201020 | He Z. |  | 308 | 20201120 | Luo X.Y. |
| 257 | 20200915 | Zeng Z.M.& Sun S.Q. |  | ~~283~~ | ~~20201020~~ | ~~Zhang D.D.~~ |  | 309 | 20201201 | Yang Z.& Chen J. |
| 258 | 20200915 | Pan D. |  | 284 | 20201021 | Li Y. |  | 310 | 20201202 | Wang M.Q.& Zheng X.D. |
| 259 | 20200916 | Cheng H.Y.& Guo X.Q. |  | 285 | 20201025 | Deng R.Y. |  | 311 | 20201203 | Chen J. et al. |
| ~~260~~ | ~~20200918~~ | ~~Wang H.& Zou J.C.~~ |  | 286 | 20201027 | Miao Z.M. et al. |  | 312 | 20201203 | Shang P. |
| 261 | 20200925 | Luo B. |  | 287 | 20201101 | Peng B.& Qin Y.M. |  | 313 | 20201205 | Deng W.Q. |
| 262 | 20200925 | Ren A.B.& Ye B. |  | 288 | 20201103 | Ma H.L. |  | 314 | 20201205 | Wang J.Y. |
| 263 | 20200925 | He J.X. |  | 289 | 20201105 | Liu K.L. |  | 315 | 20201205 | Wang Y.Y. |
| 264 | 20200925 | Liu X. |  | ~~290~~ | ~~20201105~~ | ~~Introduction~~ |  | 316 | 20201205 | Lu J.Y. |
| ~~265~~ | ~~20200925~~ | ~~Li W.M.~~ |  | 291 | 20201105 | Lin A.J.& Chen Y.X. |  | 317 | 20201207 | Wang Y.F.& Zhang C.L. |
| ~~266~~ | ~~20200925~~ | ~~Zhang J.M.& Xu Y.L.~~ |  | ~~292~~ | ~~20201110~~ | ~~Zheng X. et al.~~ |  | ~~318~~ | ~~20201209~~ | ~~Liu D. et al.~~ |
| 267 | 20200925 | Wang J.W.& Fang Z.W. |  | ~~293~~ | ~~20201110~~ | ~~Zhang S.S. et al.~~ |  | 319 | 20201210 | Wang L.& Han P.G. |
| 268 | 20200925 | Meng X.X. |  | 294 | 20201110 | He Y. |  | 320 | 20201210 | Meng Y.Y. |
| 269 | 20200925 | Cao J.F. |  | 295 | 20201110 | Zhu H.M. et al. |  | 321 | 20201210 | Liu Z.X. |
| 270 | 20200925 | Luo S.Q.& Wang S.Y. |  | 296 | 20201110 | Chen L. et al. |  | 322 | 20201210 | Yang M.Z. |
| 271 | 20200925 | Wang F. |  | 297 | 20201110 | Lu K. et al. |  | 323 | 20201215 | Zheng S.S. |
| 272 | 20200925 | Chen X.P. |  | 298 | 20201115 | Zhao W.P. |  | 324 | 20201218 | Liu X. |
| 273 | 20200926 | Yan P. |  | 299 | 20201115 | Guo J.P. |  | 325 | 20201218 | Wang J.& Ye B. |
| 274 | 20200928 | Su J. et al. |  | ~~300~~ | ~~20201115~~ | ~~Liu B.~~ |  | 326 | 20201220 | Piao Y. et al. |
| 275 | 20200929 | Mei J.M. |  | ~~301~~ | ~~20201115~~ | ~~Zhao L.L. et al.~~ |  | ~~327~~ | ~~20201225~~ | ~~Yan D.C.& Liu H.H.~~ |
| 276 | 20201001 | Zhou S. et al. |  | 302 | 20201115 | Gong C.B.& Wang Y.F. |  | 328 | 20201228 | Ye B.& Wu D.C. |
| 277 | 20201010 | Liang W.L. |  | 303 | 20201116 | Zhang J.J. |  |  |  |  |
| ~~278~~ | ~~20201010~~ | ~~Feng D.M.& Liu S.N.~~ |  | 304 | 20201118 | He J.X.& Zhang P.P. |  |  |  |  |
